# Potassium Ferrate (VI) as the Multifunctional Agent in the Treatment of Landfill Leachate

**DOI:** 10.3390/ma13215017

**Published:** 2020-11-06

**Authors:** Maciej Thomas, Violetta Kozik, Krzysztof Barbusiński, Aleksander Sochanik, Josef Jampilek, Andrzej Bąk

**Affiliations:** 1 Chemiqua Water&Wastewater Company, Skawińska 25/1, 31-066 Kraków, Poland; 2 Institute of Chemistry, University of Silesia, Szkolna 9, 40-007 Katowice, Poland; andrzej.bak@us.edu.pl; 3 Department of Water and Wastewater Engineering, Silesian University of Technology, Konarskiego 18, 44-100 Gliwice, Poland; krzysztof.barbusinski@polsl.pl; 4 Center for Translational Research and Molecular Biology of Cancer, Maria Skłodowska-Curie Memorial Cancer Center and Institute of Oncology, Wybrzeże AK 15, 44-101 Gliwice, Poland; aleksander.sochanik@io.gliwice.pl; 5 Department of Analytical Chemistry, Faculty of Natural Sciences, Comenius University, Ilkovicova 6, 84215 Bratislava, Slovakia; josef.jampilek@gmail.com

**Keywords:** landfill leachate, response surface methodology, central composite design, potassium ferrate (VI)

## Abstract

Possible use of potassium ferrate (VI) (K_2_FeO_4_) for the treatment of landfill leachate (pH = 8.9, Chemical Oxygen Demand (COD) 770 mg O_2_/L, Total Organic Carbon (TOC) 230 mg/L, Total Nitrogen (Total N) 120 mg/L, Total Phosphorus (Total P) 12 mg/L, Total Coli Count (TCC) 6.8 log CFU/mL (Colony-Forming Unit/mL), Most Probable Number (MPN) of fecal enterococci 4.0 log/100 mL, Total Proteolytic Count (TPC) 4.4 log CFU/mL) to remove COD was investigated. Central Composite Design (CCD) and Response Surface Methodology (RSM) were applied for modelling and optimizing the purification process. Conformity of experimental and predicted data (*R*^2^ = 0.8477, *R*_adj_^2^ = 0.7462) were verified using Analysis of Variance (ANOVA). Application of K_2_FeO_4_ using CCD/RSM allowed to decrease COD, TOC, Total N, Total P, TCC, MPN of fecal enterococci and TPC by 76.2%, 82.6%, 68.3%, 91.6%, 99.0%, 95.8% and 99.3%, respectively, by using K_2_FeO_4_ 0.390 g/L, at pH = 2.3 within 25 min. Application of equivalent amount of iron (as FeSO_4_ × 7H_2_O and FeCl_3_ × 6H_2_O) under the same conditions allowed to diminish COD, TOC, Total N, Total P, TCC, MPN of fecal enterococci and TPC only by 38.1%, 37.0%, 20.8%, 95.8%, 94.4%, 58.2%, 90.8% and 41.6%, 45.7%, 29.2%, 95.8%, 92.1%, 58.2%, 90.0%, respectively. Thus, K_2_FeO_4_ could be applied as an environmentally friendly reagent for landfill leachate treatment.

## 1. Introduction

Several aspects of human agricultural and industrial activity are related to adverse changes in the quality of water resources worldwide. Undoubtedly, such activity has a direct impact on the waste production. In fact, some waste undergoes various disposal processes while other is deposited in municipal landfills. As a result of the municipal waste landfills exploitation, leachate is generated. In case of insufficient protection, it may get into the soil or groundwater and, due to its physicochemical and microbiological composition, this may significantly contribute to the groundwater contamination. The amount of leachate and their characteristics depend on a number of factors, including: type of waste, degree of fragmentation, compaction and storage method, landform, amount of precipitation, method of sealing the bottom of the landfill, type of vegetation covering the landfill, soil conditions, etc. [1]. It is widely known that the values of parameters (e.g., COD, TOC etc.) for the landfill leachate were higher in the dry season than in the rainy season for the fresh leachate samples. Recent research points to a higher content of heavy metals in the suspensions. Moreover, there were no significant seasonal changes in the concentration of heavy metal ions in suspended solids and sediment samples [2].

Nevertheless, depending on the age of the landfill (young < 5 years; medium 5–10 years; old > 10 years), the physicochemical composition of the leachate may vary [3,4]. According to the available data [4,5,6,7,8,9,10,11,12], the pH-value of the leachate varies in the range of 4–7.6, 6.9–9 and 8.1–9.5 for the leachate from young, medium and old landfills, respectively. In the case of COD and TOC, a decrease in the values of these indicators was observed according to the age of the landfills, and for COD they amounted to approx. 1.87–84.30, 0.56–9.50 and 0.10–3.46 g O_2_/L [4,13,14,15,16], and for TOC approx. 1.60–13.61 g/L, 0.19–2.05 g/L and 0.04–1.90 g/L [16,17,18,19,20,21,22,23]. Similar dependencies were observed in the case of total nitrogen (approx. 1.75–4.37, 0.35–3.00 and 0.42–2.64 g/L) and total phosphorus (approx. 2–655, 3–18 and 1–7 mg/L) [18,24,25,26,27,28,29,30,31]. Municipal landfills effluents may also contain small amounts of heavy metals such as: Cd (<0.02–6.5 mg/L), Cu (0.005–6 mg/L), Pb (0.01–3.50 mg/L), and even Cr^6+^ (0.04–8.4 mg/L) [4,31,32,33,34].

Due to the fact that the leachate from municipal landfills comprises a significant amount of organic compounds and shows significant physicochemical parameters variation depending on their age, they create many technical and technological problems during their treatment. For this reason, various physicochemical and biological methods are used to treat the leachate. The landfill leachate could be treated with: ozone after coagulation treatment [7], ozonation [21], hydrodynamic cavitation [11], catalytic oxidation (by using Ni/Al_2_O_3_ as the catalyst) by supercritical water oxidation [12], coagulation-flocculation, chemical coagulation and reverse osmosis system [13,30], hybrid coagulation-nanofiltration process [20], electrocoagulation [15], electrochemical oxidation [17], photoelectrochemical treatment in a continuous flow reactor [16], coagulation and Fenton reagent, UV, H_2_O_2_ and UV/H_2_O_2_ process [35,36,37].

The presented physicochemical methods require the use of physical factors (e.g., pressure, electric current etc.), various chemical compounds (coagulants, catalysts, etc.) devices and conditions in order to obtain high purification efficiency. Not all of these methods have found practical application due to, sophisticated technical solutions or complicated cleaning procedures implemented.

The results reported in literature indicate that the treatment of leachate requires biological procedures with activated sludge [38,39] and even phytoremediation methods [31]. Biological methods are characterized by varying effectiveness, which is related to higher biodegradability of leachate from young landfills compared to the old ones (reduction in the BOD_5_/COD ratio where BOD_5_ is five-day Biochemical Oxygen Demand) [40]. Therefore, landfill leachate introduced into biological wastewater treatment plants may have a negative influence on the microorganisms of the activated sludge and reduce the treatment efficiency. In particular, some chemical compounds present in landfill leachate, such as chlorobenzene, dichlorobenzene, chlorophenols, chloroaniline, toluene, ethylbenzene, xylene, phthalates and polycyclic aromatic hydrocarbons (PAHs) [40,41] have a negative influence on the activity of activated sludge. For this reason, direct application of biological methods for leachate treatment before prior implementation of physicochemical methods is not always possible. Biological methods seem to be the most environmentally friendly, unfortunately, may show variable effectiveness due to changing concentrations of pollutants in the treated leachates.

Currently, more and more attention is being paid to such methods of leachate treatment which are environmentally friendly and do not cause additional negative effects. This concept is ideally suited to the method using potassium ferrate (VI) (K_2_FeO_4_), which is an eco-friendly powerful oxidant with a dual mechanism of action. On the one hand, it acts in the oxidation of organic and partially inorganic pollutants (simultaneous reduction of Fe^6+^ to Fe^3+^), and in the coagulation of pollutants or oxidation products and their adsorption on the flocs of hydrated Fe(OH)_3_. Due to the fact that the decomposition products of K_2_FeO_4_ are iron oxides and oxygen, it is defined as a green oxidant and can be a promising alternative to the conventional coagulants [42]. Among others, potassium ferrate (VI) has been engaged for degradation of endocrine-disrupting compounds (EDCs), decomposition of surfactants (SPCs), personal care products (PCPs), pharmaceuticals [43], and also for oxidation of cyanides (CN^−^) originating from the mining and processing of gold ore, degradation of natural organic matter (NOM), oxidation of N,N–diethyl–3–toluamide (DEET), many dyes (Methylene Blue, Orange II, Brilliant Red X-3B, Acid Green 16), removal of algae [44] and for wastewater treatment [45].

The principal objective of the presented study was to assess the possibility of using K_2_FeO_4_ for the treatment of leachate from a municipal waste landfill and to select the most favorable conditions (pH, K_2_FeO_4_ conc., reaction time) for the treatment of leachate ensuring the maximum reduction of the COD value. Comparative studies were also carried out with the use of conventional coagulants (FeSO_4_ × 7H_2_O, FeCl_3_ × 6H_2_O) containing an equivalent amount of iron (in relation to the amount contained in the most favorable dose of K_2_FeO_4_) and the effect of the iron salts used on the concentration of Total Coli Count (TCC), Most Probable Number of fecal enterococci (MPN) and Total Proteolytic Count (TPC) in the treated leachate.

## 2. Materials and Methods

### 2.1. Chemicals

Envifer^®^ (Nano Iron, Zidlochovice, Czech Republic) was engaged as the K_2_FeO_4_ source. Due to its chemical instability the content of K_2_FeO_4_ in Envifer^®^ was specified directly before the procedures provided in the Analytical Procedures section. Envifer^®^ was entirely characterized (UV-VIS spectrum, energy-dispersive X-ray spectroscopy (EDXS) analysis, scanning electron microscopy (SEM) analysis) previously [46]. To adjust the leachate sample pH, 5% and 20% solutions of H_2_SO_4_ (Avantor^TM^, Gliwice, Poland) was used. Solid FeSO_4_ × 7H_2_O and FeCl_3_ × 6H_2_O (Chempur, Piekary Śląskie, Poland) were applied as conventional coagulants. For sludge flocculation a 0.05% solution of anionic flocculant Furoflock CW277 (Chemische Fabrik Wocklum Gebr. Hertin GmbH&Co. KG, Balve, Germany) was employed. All chemicals (except K_2_FeO_4_) were of analytical grade. Additionally, deionized water (<2 µS/cm) was used for preparation and dilution of the solutions.

### 2.2. Origin and Physicochemical Parameters of the Landfill Leachate

Leachate from the old (>10 years) municipal waste landfill located in southern Poland was investigated. The effluents were collected in summer during the rainy season (air temperature 25 ± 1 °C, precipitation 8 mm of water column) from the effluent reservoir, where it flowed through a system of drainage pipes. Fresh leachate (inflow) was collected within 24 h, every hour, into sterile 1 L bottles, which were stored at the temperature of 4 ± 1 °C without fixing the leachate before further investigation. The average sample of the leachate used for the test was obtained by mixing 1 L of unit samples in a sterile 25 L canister. The raw landfill leachate was analyzed as described in the Analytical Procedures section.

### 2.3. Apparatus and Experiment Conditions

All experiments were conducted at a constant temperature (19 ± 1 °C), in beakers containing 250 ± 1 mL of tested leachates. The samples were mixed using a magnetic stirrer (MS11, Wigo, Pruszków, Poland) at a constant speed of 250 rpm at the oxidation/coagulation stage and 50 rpm at the flocculation stage. The experiments with K_2_FeO_4_ were carried out in such a way that K_2_FeO_4_ was added to the measured volume of wastewater, the pH was corrected with 20% H_2_SO_4_ and the reaction was carried out for the assumed time span. The quantity of K_2_FeO_4_, pH and reaction time were set as predetermined at the stage of planning the experiments. After the oxidation/coagulation process was completed, the pH was adjusted to 9.0 ± 0.1 in each experiment using 20% NaOH in order to precipitate the Fe^3+^ ions as Fe(OH)_3_. Subsequently, 0.25 mL of 0.05% Furoflock CW277 solution (anionic flocculant) was added, and the stirring speed was decreased to 50 rpm. After 30 sec, stirring was halted in order to sediment the formed precipitate. A sample of the liquid above the precipitate was collected and filtered using a 0.45 µm PTFE syringe filter before COD determination. The filtrate was analysed according to the procedure provided in the Analytical Procedures section. Under the most favorable conditions of reducing the COD value (pH, K_2_FeO_4_ conc., reaction time), a verification experiment was carried out using sterile glass and laboratory equipment. In this case, a sample of treated leachate above the sediment was collected without filtering it through a 0.45 µm PTFE syringe filter and microbiological tests were performed. Under the same conditions (pH, reaction time), comparative tests were performed using an equivalent iron dose (K_2_FeO_4_ vs. FeSO_4_ × 7H_2_O and FeCl_3_ × 6H_2_O). In each case the treated leachate was analyzed as described in the Analytical Procedures section.

### 2.4. Analytical Procedures

Before performing the tests the chromite titration method was engaged to specify the content of K_2_FeO_4_ in Envifer^®^. The above method is composed of oxidizing Cr(OH)_4_^−^ ions using FeO_4_^2−^ under extremely alkaline conditions, which results in the generation of Fe(OH)_3_, CrO_4_^2−^, OH^−^. The K_2_FeO_4_ content in technical grade product (%) was calculated using the following formula:

(1) $$\%\;of\;K_{2}FeO_{4}=\;\frac{c_{Fe\left(II\right)}\;\times V_{Fe\left(II\right)}\;\times M_{K_{2}FeO_{4}}\;\times 100\%}{3000\;\times \;m_{sample}}$$

where $c_{Fe\left(II\right)}$ and $V_{Fe\left(II\right)}$ are the concentration (0.0850 mol/L) and the volume (mL) of the standard Mohr’s salt (ammonium iron(II) sulphate, (NH_4_)_2_Fe(SO_4_)_2_) solution, $M_{K_{2}FeO_{4}}$ is 198.04 g/mol, and $m_{sample}$ represents the sample weight (g) [47]. The determination of the K_2_FeO_4_ content in Envifer^®^ was also carried out spectrophotometrically (Cary^®^ 50 UV-VIS, Varian Inc., Melbourne, Australia) [48]. In this case, an Envifer^®^ sample (with accuracy ± 0.001 g) was dissolved in deionized water, and the volume was adjusted to 100 mL in a volumetric flask. Subsequently, the sample was filtered (0.45 µm) into a quartz cuvette (light path = 10 mm) and the absorbance values at λ = 505 nm was measured instantaneously. The K_2_FeO_4_ content in Envifer^®^ (%) was specified according to the following formula:

(2) $$\%\;of\;K_{2}FeO_{4}=\;\frac{A\;\times 0.1\;\times M_{K_{2}FeO_{4}}\;\times 100\%}{1070\;\times \;m_{sample}}$$

where *A* is the absorbance at 505 nm, $M_{K_{2}FeO_{4}}$ is 198.04 g/mol, 1070 is the molar absorbance coefficient, M^−1^ cm^−1^, and $m_{sample}$ represents the sample weight (g).

The pH-values and temperature were measured using an Inolab^®^ pH/Ion/Cond/Temp 750 m and SenTix^®^ 81 electrodes (WTW, Weilheim in Oberbayern, Germany) [49]. The landfill leachate COD values were evaluated employing a dichromate method and the PF-11 spectrophotometer [50]. TOC was assayed using the tube test kit Nanocolor^®^ TOC 60, while the end-point was specified using the PF-11 spectrophotometer. TOC assessment was conducted in two stages. In the first one, inorganic carbon was eradicated from the samples by adding NaHSO_4_ and stirring the sample (500 rpm, 10 min). In the second stage, organic compounds were degraded by application of Na_2_S_2_O_8_ at 120 °C for 120 min, and thymol blue absorbance variations of sodium salt solution were measured spectrophotometrically at λ = 585 nm [51]. Determination of Total Nitrogen (TN) was performed by two-step spectrophotometric method using Nanocolor^®^ Total Nitrogen 220 test tube kit (Macherey-Nagel, Düren, Germany). In the first stage, the wastewater sample was mineralized (Na_2_S_2_O_8_, H_2_SO_4_, 120 °C, 30 min), and in the second stage, spectrophotometric determination of nitrogen compounds after their reaction with 2,6–dimethylphenol (DMP, also commonly known as 2,6–xylenol), in a mixture of H_2_SO_4_ and H_3_PO_4_ were carried out [52]. Determination of Total Phosphorus (TP) was performed after effluent sample mineralization (Na_2_S_2_O_8_, H_2_SO_4_, 120 °C, 30 min) by using a test tube kit Nanocolor^®^ ortho– and total Phosphate 15, with spectrophotometric endpoint detection using a PF-11 apparatus (Macherey-Nagel, Düren, Germany) [53]. The dilution of leachate samples before microbiological enumeration was performed according to ISO 6887-1:2017 [54]. The enumeration of Total Coli Count (TCC, CFU/mL), Total Proteolytic Count (TPC, CFU/mL) and the Most Probable Number of faecal enterococci (MPN/100 mL) were determined according to ISO 4832:2006 [55], PN-75/C-04615/17:1975 [56] and PN-C-04615-25:2008 [57], respectively. For the precipitation of gelatin in the Frazier’s medium, Frazier’s reagent (the mixture of HgCl_2_, HCl and H_2_O) was used.

### 2.5. Response Surface Methodology

Central Composite Design (CCD) and Response Surface Methodology (RSM) were engaged to specify the most favorable conditions for lowering the COD landfill leachate value. The optimization of the COD removal process consisted of determining the numerical values of three independent variables (pH, K_2_FeO_4_ dose and reaction time) for which the value of the dependent parameter (COD) was the lowest. Based on the literature data on the implementation of K_2_FeO_4_ for the treatment of wastewater from various sources and taking into account the value of the redox potential for the FeO_4_^2−^ ion (E° = +2.20 V in acidic and E° = +0.72 V in neutral media) and own experience, several preliminary experiments were carried out. The results of these experiments made it possible to approximate the pH, K_2_FeO_4_ and reaction time adopted at the stage of planning experiments with the use of CCD. Therefore, the following values of input parameters were investigated: pH in the range 2–6, K_2_FeO_4_ dose 0.2–0.4 g/L and reaction time 10–20 min. The values of the remaining variables, including i.e., temperature, stirring speed, and volume of the treated wastewater sample were set as constant in each experiment, respectively. Table 1 reports the set-up of the 16 experiments designated by using CCD.

The obtained empirical findings (the arithmetic mean of three runs was adopted) were investigated statistically; and the impact of the independent (input) variables (pH, concentration of K_2_FeO_4_ (g/L), and reaction time (min)) on the dependent (output) parameter (COD, g O_2_/L) was illustrated as a response surface graph. For the most favorable values of the three input parameters, an experimental verification of the model was carried out (additionally, the COD changes after 25 min, 30 min, 35 min and 40 min reaction time were investigated).

## 3. Results and Discussion

### 3.1. Physicochemical Parameters of the Landfill Leachate and K_2_FeO_4_

The physicochemical analysis of technical grade potassium ferrate (VI) showed that it contained 40% of pure K_2_FeO_4_. Additionally, previous research revealed that it was composed of 47.31% ± 1.50% K, 15.00% ± 0.45% Fe, and 37.69% ± 5.20% O, along with impurities such as K_2_O and ferrous compounds other than K_2_FeO_4_ (i.e., K_3_FeO_4_ and KFeO_2_). It was probably related to the method used at the stage of its synthesis [46].

Table 2 presents chosen physicochemical and microbiological variables of the landfill leachate.

Initiatory specification of the chosen physicochemical and microbiological parameters of the landfill leachate unveiled that they were slightly alkaline (pH = 8.9) and contained a certain amount of organic compounds expressed as chemical oxygen demand and total organic carbon (COD 770 mg O_2_/L and TOC 230 mg/L, respectively). Additionally, the content of organic (and probably inorganic) nitrogen compounds in the tested leachates was specified by the content of total nitrogen and phosphorus (TN 120 mg/L and TP 12 mg/L, respectively).

On the other hand, the conducted microbiological tests showed significant contamination of the investigated leachates with coliforms, fecal bacteria and proteolytic bacteria (TCC 6.8 log CFU/mL, MPN 4.0 log/100 mL and 4.4 log CFU/mL, respectively). The obtained test results are comparable with the previous findings, especially for leachate from old landfills, for which a decrease in the value of pollution indicators was observed. Generally, in the case of pH-value values were 4–9.5 [4,5,6,7,8,9,10,11,12], for COD 100–84 300 mg O_2_/L [4,13,14,15,16] and for TOC 40–13 610 mg/L. In addition, for TN and TP, the values were 350–4.370 mg/L and 1–655 mg/L, respectively [18,24,25,26,27,28,29,30,31]. The parameter values presented in Table 2 indicate that the tested leachate did come from the old landfill and was collected during the rainy season, as presented in the section concerning origin and physicochemical parameters of the landfill leachate. Other studies indicated the presence of pathogenic bacteria, not only in the leachate, but also in groundwater as a result of leachate infiltration into the ground (coliform bacteria, *Escherichia coli*, *Enterococci*, *Pseudomonas aeruginosa*). In groundwater, high concentrations of coliform bacteria (20,000 CFU/100 mL), *Escherichia coli* (15,199 CFU/100 mL) and *Enterococci* (3290 CFU/100 mL) were specified [58]. The conducted studies of leachate revealed that in the event of their uncontrolled release, they may have a negative impact on the natural environment.

### 3.2. CCD/RSM Findings

The employment of CCD and RSM in investigation planning enabled 16 experiments to be performed (see Table 2). The findings of COD values (g O_2_/L) linked to each experiment are reported in Table 2 (see column 5). The lowest COD values (<0.25 g O_2_/L) were recorded in experiments 9, 12, and 14 (0.245, 0.205, 0.240 g O_2_/L), respectively. In the experiment number 12, the highest dose of K_2_FeO_4_ (0.468 g/L) was used at pH 3.5 during 15 min, and the lowest COD value was obtained for the purified effluents (0.205 g O_2_/L). This indicates a significant influence of the K_2_FeO_4_ dose on the COD value of the effluents, along with other parameters (pH-value and reaction time).

Table 3 presents the evaluation of the parameters and their influence of the COD of the landfill leachate.

The constant value, pH (L), K_2_FeO_4_ (L) and K_2_FeO_4_ (Q), concentration were specified to be statistically valid (*p* < 0.05), while the pH (Q), Time (L) and Time (Q) were not statistically significant (*p* > 0.05). Moreover, the values of the calculated determination coefficient R^2^ and the adjusted determination coefficient R^2^_adj_ (0.8477 vs. 0.7462) depicted the ratio of the variance in the dependent variable (COD) that was foreseen based on the independent variables (pH -value, K_2_FeO_4_ conc. and time).

In the case of the real sewage from the textile industry, R^2^ and R^2^_adj_ reached values of 0.8799 and 0.7999 [45]. In the case of using K_2_FeO_4_ for the treatment of wastewater from the tanning industry other studies have reported R^2^ and R^2^_adj_ values of 0.77 and 0.59 [59] versus 0.95 and 0.74 in the case of employing K_2_FeO_4_ for the treatment of synthetic sewage containing azo dye Anilan Blue GRL 250% [60]. A good fit between the empirical and approximated data was observed in the latter study.

**Figure 1:**
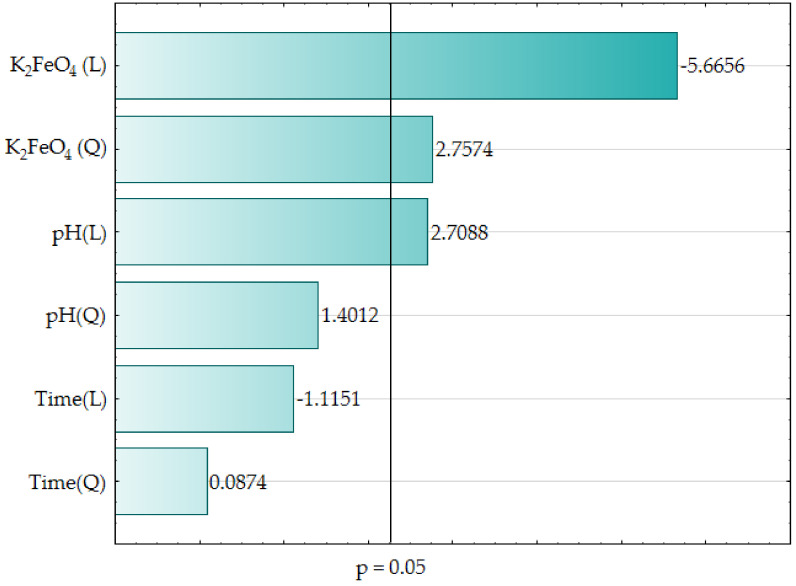
Bar chart of standardized effects (COD, g O_2_/L, 3 value, 1 block, 16 experiments, MS = 0.0040, L–linear effect, Q–quadratic effect, p–the absolute value of the standardized effect evaluation).

Table 4 reports the outcome of verifying the adequacy of the model coefficients using ANOVA, which confirmed the statistical significance (*p* < 0.05) of the main input parameters i.e., pH (L), K_2_FeO_4_ (L) and K_2_FeO_4_ (Q). These findings are also presented graphically in a form of bar chart (see Figure 1).

**Figure 2:**
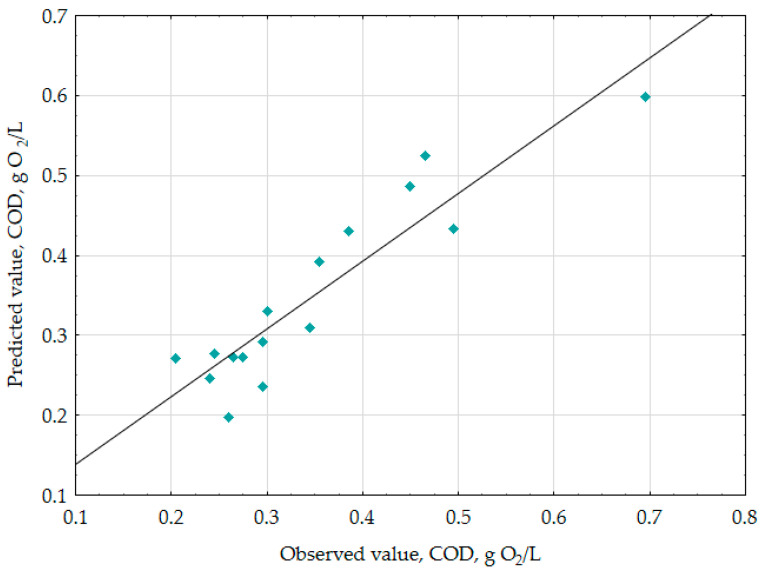
Predicted vs. observed values plots for COD (g O_2_/L).

The estimators of the standardized effects were prioritized according to their absolute value; the vertical line pinpoints the minimum absolute value for statistical significance. In the investigated wastewater samples, K_2_FeO_4_ (L), K_2_FeO_4_ (Q), and pH (L), revealed the largest impact on decreasing the COD value under the empirical conditions. The other parameters i.e., pH (Q), Time (L), time (Q) exerted the smallest impact on the COD value. Figure 2 presents the relationship between the predicted COD value and observed COD value.

**Figure 3:**
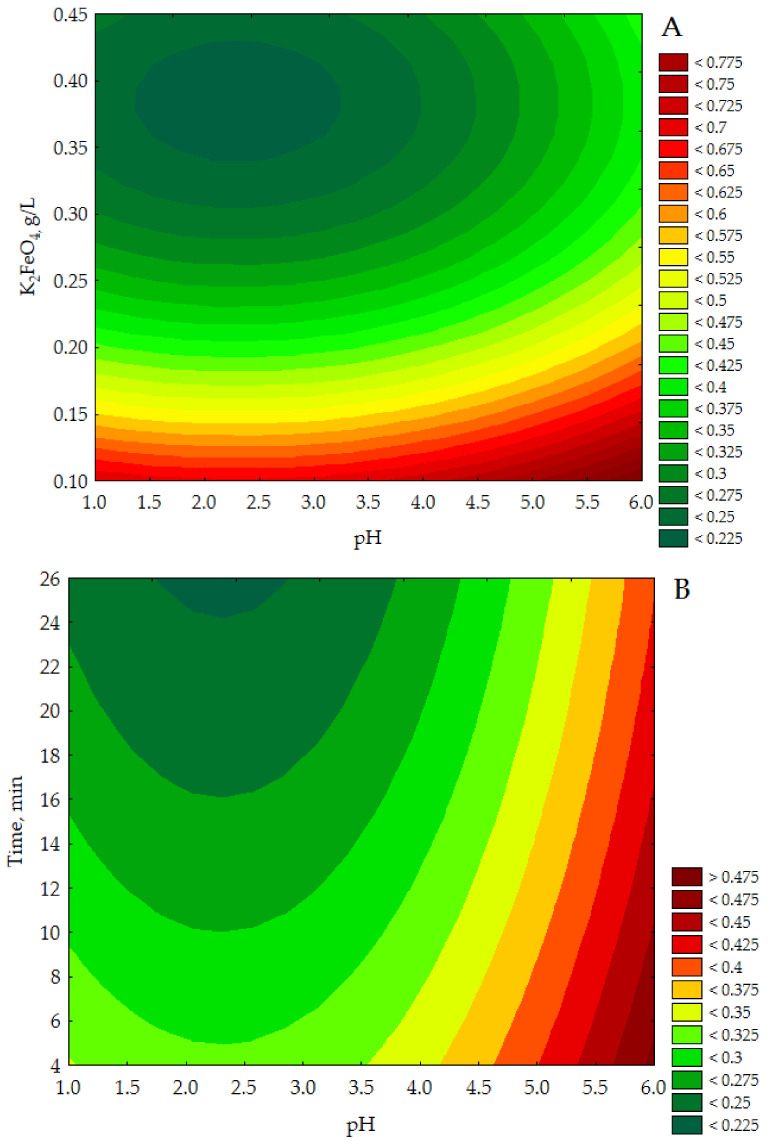

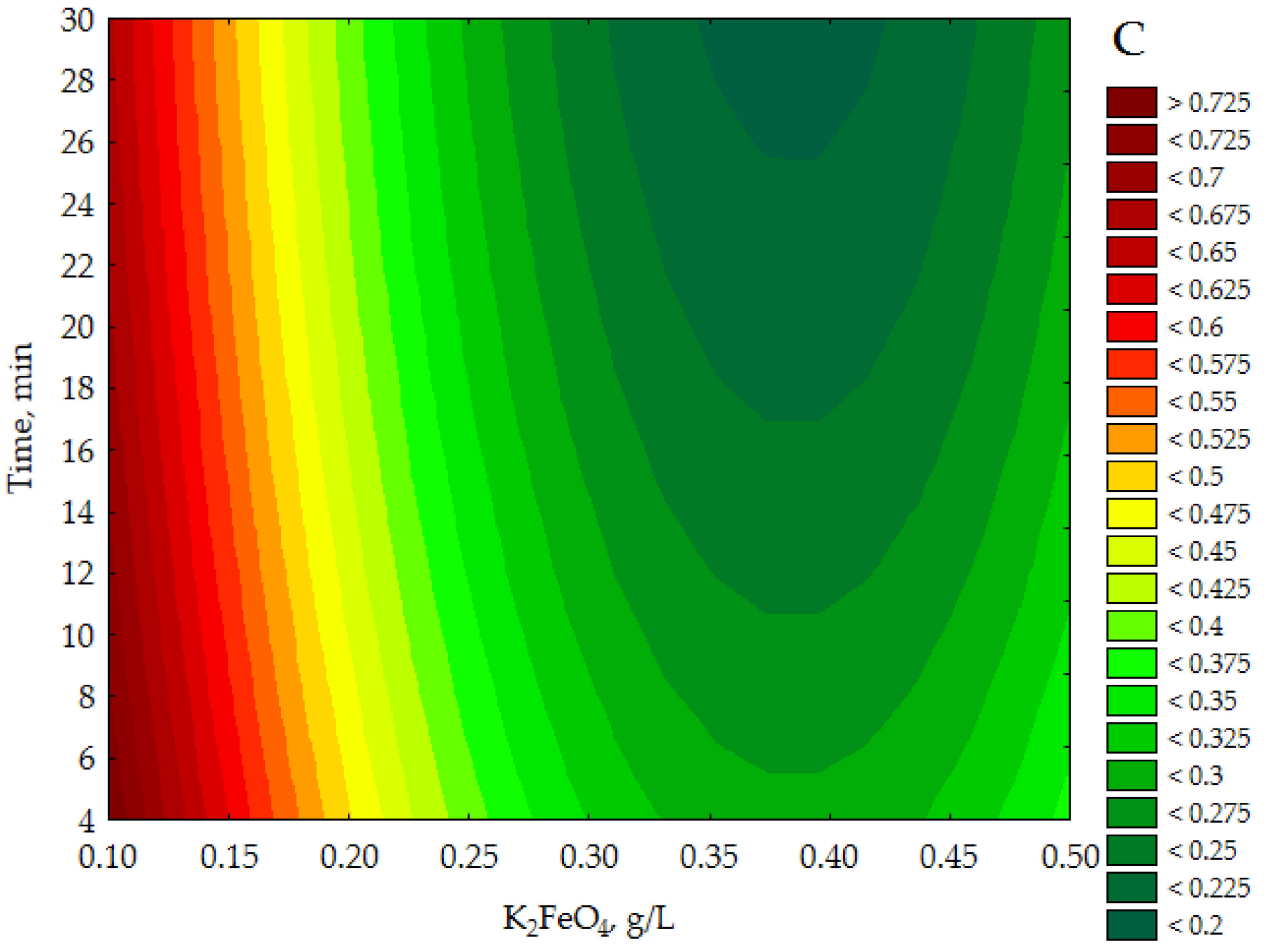
Response surface plots for COD (g O_2_/L) with respect to K_2_FeO_4_ (g/L) and pH (**A**), time (min), and pH (g/L) (**B**) and time (min) and K_2_FeO_4_ (g/L) (**C**).

The data presented a linear correlation between the empirical and approximated data in the range of verified COD values. Figure 3 illustrates the response surface plots for COD with respect to K_2_FeO_4_ conc. and pH, Time and pH and Time and to K_2_FeO_4_ conc. (see Figure 3A–C).

The CCD/RSM study showed (see Figure 3A) that the lowest COD value (<0.225 g O_2_/L) was obtained for K_2_FeO_4_ approx. 0.34–0.43 g/L and a pH between 1.4–3.3 with the time parameter set at 15 min. It can be seen in Figure 3B that for a constant dose of K_2_FeO_4_ 0.300 g/L the lowest COD values (<0.225 g O_2_/L) were specified for pH approx. 1.7–2.9 in more than 25 min. In turn, the data presented in Figure 3C indicate that the adoption of a constant pH value of 3.5 allows to obtain the lowest COD values of purified effluents (<0.200 g/L) for K_2_FeO_4_ conc. approx. 0.35–0.43 g/L over a time greater than 25 min. The presented results of model tests show that the lowest COD values for purified leachates were determined for the highest doses of K_2_FeO_4_ used in the experiments, in the acidic environment (1.7 < pH < 3.3) for more than 25 min.

The generated test results correspond to the literature data, which indicate that the value of the redox potential for the FeO_4_^2−^ ion is greater in an acidic environment than in a neutral environment (E° = +2.20 V in acidic and E° = +0.72 V in neutral media). Generally, greater efficiency of the oxidation of organic compounds can be observed, while conducting the oxidation process in an acidic environment, rather than in a neutral one [43,44]. Another study indicated that use of K_2_FeO_4_ for the purification of highly polluted tannery wastewater from leather dyeing processes resulted in the discoloration (98.4% removal), chemical oxygen demand (77.2% removal), total organic carbon (75.7% removal), and suspended solids (96.9% removal); the reported values were the smallest when 1.200 g/L K_2_FeO_4_ at pH 3 within 9 min was used [59]. On the other hand, the application of K_2_FeO_4_ for the degradation of trichloroacetic acid and turbidity removal in synthetic water revealed that the highest efficiency achieved for trichloroacetic acid was 24%, while for turbidity the maximum removal efficiency was in the range of 85%–95%. Additionally, the optimum conditions for initial turbidity, pH, and ferrate (VI) dosage were 8.89 NTU, 3, and 4.26 mg/L as Fe, respectively [61]. Other study indicates that the leachate treatment is also possible in an alkaline condition. In this case the pH value was 10, the dosage of K_2_FeO_4_ was 6 g/L and the reaction time was 30 min. Unfortunately, the experiment required an additional use of a stabilizer at a dose 4 g/L (sodium silicate, Na_2_SiO_3_). Under those conditions, the COD removal efficiency was only 36% [62] compared to 76.2% in this study. An application of K_2_FeO_4_ in the leachate treatment at the higher temperature (30 °C) by the initial ferrate (VI) to COD mass concentration ratio of 0.50, pH 4.00 and reaction time 40 min was suggested as well. It was stated that the leachate from hazardous waste landfill which was pretreated by K_2_FeO_4_ could be directly discharged into the biological treatment system. However, COD value of leachates from the refuse incineration plant which was pretreated by K_2_FeO_4_ was as much as 2861 mg O_2_/L. These leachates required re-treatment before the introduction into the subsequent biochemical treatment system [63].

Moreover, it should be taken into account that the total efficiency of removing organic (and partially inorganic) compounds expressed as COD, TOC, TN and TP results not only from their oxidation by Fe^+6^, but also to some extent from their adsorption on freshly precipitated Fe(OH)_3_ flocs with a large active surface. In the case of phosphorus compounds (present as PO_4_^3−^), it is possible to remove them by co-precipitation with Fe^3+^ ions, which results in the formation of hardly soluble ferric phosphate. To sum up, it should be stated that under the experimental conditions, the total efficiency of removing contaminants expressed as COD resulted from their oxidation and coagulation and, probably, to some extent from adsorption and co-precipitation. Table 5 presents the calculated coefficients of the fitted model.

Consequently, the changes in the COD value can be calculated according to the following formula:

COD (g O_2_/L) = 1.206468−0.059897(pH) + 0.012988(pH)^2^ − 4.423640(K_2_FeO_4_) + 5.750741 (K_2_FeO_4_)^2^ − 0.006018(Time) + 0.000073(Time)^2^ (3)

For the most favorable values of the three input parameters (pH = 2.31, K_2_FeO_4_ 0.38 g/L and Time 41 min) calculated from the model, the estimated COD value was 162 mg O_2_/L. In the conducted verification experiment, the COD value was 178 mg O_2_/L. Assuming that the uncertainty of COD determination is ± 15%, the actual COD value of sewage treated under the most favorable conditions is in the range from 151 to 205 mg O_2_/L (180 ± 27 mg O_2_/L), which also includes the estimated value from model for the most favorable pH values, K_2_FeO_4_ and time.

For a constant pH value 3.5 (see Figure 3B) the lowest COD values (<200 mg O_2_/L) were obtained after 25 min of reaction time, therefore an additional verification experiment was carried out for the pH value and K_2_FeO_4_ concentration estimated from the model for the most favorable conditions (i.e., 2.31 g/L and 0.38 g/L, respectively), and the COD was determined after 25 min, 30 min, 35 min and 40 min reaction time. The subsequent COD values of the treated effluents were 180 ± 27 mg O_2_/L, 172 ± 26 mg O_2_/L, 170 ± 26 mg O_2_/L, 168 ± 25 mg O_2_/L, respectively. Considering the uncertainty of the COD determination (± 15%), it was found that the COD of the treated leachate was not significantly reduced. Therefore, the most favorable values for the independent parameters i.e., pH = 2.3 ± 0.1, K_2_FeO_4_ 0.390 ± 0.001 g/L and Time 25 ± 1 min were adopted. Under these conditions, a reduction in TOC, TN and TP was also observed (82.6%, 68.3%, 91.6%, respectively) as shown in Table 6 (column 3).

### 3.3. Coagulation/Flocculation Findings and K_2_FeO_4_ Biocidal Properties

Table 6 reports the findings of tests of purified leachates after the application of K_2_FeO_4_ (under optimal conditions) and FeSO_4_ × 7H_2_O and FeCl_3_ × 6H_2_O in an amount equivalent to the dose of iron contained in 0.390 g of K_2_FeO_4_.

The test results revealed that the use of an equivalent dose of iron in the form of FeSO_4_ × 7H_2_O and FeCl_3_ × 6H_2_O made it possible to reduce the COD, TOC, TN values only by 38.1%, 37.0%, 20.8% (in the case of FeSO_4_ × 7H_2_O) and 41.6%, 45.7%, and 29.2% (in the case of (FeCl_3_ × 6H_2_O), compared to K_2_FeO_4_, the application of which was much more effective (see Table 6, column 3). Additionally, in all cases a reduction of the TP value > 90% was achieved. It is clear that the removal of impurities from the tested leachates was not only due to coagulation, co-precipitation and adsorption (as in the case with conventional coagulants), but also as a result of oxidation process using K_2_FeO_4_. Additionally, it should be noted that in the case of using conventional coagulants, the efficiency of removing microorganisms was comparable and amounted to 92.1%, 58.2%, 90.0% (in the case FeCl_3_ × 6H_2_O for TCC, MPN, TPC) and 94.4%, 58.2%, 90.8% (in the case FeSO_4_ × 7H_2_O for TCC, MPN, TPC), respectively. When K_2_FeO_4_ was used the microorganism removal efficiency was 99.9%, 95.8% and 99.3% for TCC, MPN and TPC, respectively.

The obtained results indicate an additional biocidal effect related to the oxidizing properties of the FeO_4_^2−^ ion in an acidic environment. Other studies revealed that K_2_FeO_4_ can reach the disinfection targets (>6 log inactivation of *Escherichia coli*) at a very low dose (6 mg/L as Fe) and over wide working pH range compared to chlorination (10 mg/L as Cl_2_) and coagulation (Fe_2_(SO_4_)_3_ 3.4 mg/L as Fe). In wastewater treatment, K_2_FeO_4_ kill 3 log more bacteria in comparison with Al_2_(SO_4_)_3_ and Fe_2_(SO_4_)_3_ at a similar or even smaller dose [64]. Reports have shown that ferrate (VI) has excellent disinfectant properties and can inactivate a wide variety of microorganisms at low ferrate (VI) dosages. Additionally, ferrate (VI) can disable many chlorine-resistant organisms, such as aerobic spore-formers and sulphite-reducing *Clostridia*. The ferrate (VI) can deactivate not only *Escherichia coli* at lower dosages or shorter contact time than hypochlorite, but also *Bacillus cereus, Streptococcus bovis, Staphylococcus aureus, Shigella flexneri, Streptococcus faecalis* and *Salmonella typhimurium*, respectively. In turn, ferrate (V) has been proven to be highly reactive and about 10^3^–10^5^ times more reactive to impurities than ferrate (VI), suggesting that the eradication of toxins by ferrate (VI) may be enhanced in the presence of appropriate one-electron-reducing agents. The ferrate (V) has the capability of inactivating biological species and toxins, which cannot be reached by ferrate (VI) [65]. Since the high reactivity of ferrate (V) allows to inactivate biological species and toxins which cannot be eliminated by ferrate (VI), it seems that this property may also be responsible for inactivation of bacteria.

Moreover, recent investigations suggested that iron sludge containing iron (III) salts and hydroxides that left after the treatment of leachate may be reused for manufacturing of ferrate (VI) [66]. This possibility of reusing sludge after treatment fits very well to the concept of a circular economy. From the practical and technological point of view, it is important to be able to generate ferrate (VI) in situ [67], which reduces the costs of synthesis, transport, storage and handling.

## 4. Conclusions

The use of potassium ferrate (VI) for the treatment of leachate from a municipal landfill site made it possible to obtain clean leachate characterized by low values of physicochemical (COD, TOC, TN, TP) and microbiological (TCC, MPN, TPC) parameters. Under optimal conditions, potassium ferrate (VI) effectively decomposed organic compounds present in the leachate and inactivated microorganisms, which was related to its disinfecting effect. The use of conventional coagulants in the form of iron (II) and (III) salts allowed for only partial removal of impurities from the tested leachate. Both in the case of potassium ferrate (VI) and conventional coagulants, iron (II) and (III) hydroxides are formed, which can adsorb impurities or lead to their co-precipitation. The maximum efficiency of pollutant removal was obtained with the use of K_2_FeO_4_ in the process of their oxidation, and then coagulation, adsorption and co-precipitation. Moreover, iron sludge left after the treatment of leachate may be reused for generation of ferrate. Thus, K_2_FeO_4_ can be treated as an effective, multi-functional and environmentally-friendly coagulant for the treatment of leachate from municipal landfills.

## Figures and Tables

**Table 1:** Empirical conditions for the CCD/RSM and outcome (COD) for landfill leachate (pH 0.98–6.02, K_2_FeO_4_ 0.132–0.468 g/L, Time 6.59–23.41 min); (C)-center of the plan.

Run	Experimental Conditions			Experimental Results *
	pH	K_2_FeO_4_ (g/L)	Time (min)	COD (g O_2_/L)
1	2.00	0.200	10.00	0.385 ± 0.058
2	2.00	0.200	20.00	0.355 ± 0.053
3	2.00	0.400	10.00	0.295 ± 0.044
4	2.00	0.400	20.00	0.260 ± 0.039
5	5.00	0.200	10.00	0.465 ± 0.070
6	5.00	0.200	20.00	0.450 ± 0.068
7	5.00	0.400	10.00	0.300 ± 0.045
8	5.00	0.400	20.00	0.295 ± 0.044
9	0.98	0.300	15.00	0.245 ± 0.037
10	6.02	0.300	15.00	0.495 ± 0.074
11	3.50	0.132	15.00	0.695 ± 0.104
12	3.50	0.468	15.00	0.205 ± 0.031
13	3.50	0.300	6.59	0.345 ± 0.052
14	3.50	0.300	23.41	0.240 ± 0.036
15 (C)	3.50	0.300	15.00	0.265 ± 0.040
16 (C)	3.50	0.300	15.00	0.275 ± 0.041

* parameter value ± the measurement uncertainty for an extension factor *k* = 2.

**Table 2:** The specified physicochemical and microbiological parameters of the landfill leachate.

Parameter	Unit	Result *
pH	–	8.9 ± 0.1
Chemical Oxygen Demand, COD	mg O_2_/L	770 ± 116
Total Organic Carbon, TOC	mg/L	230 ± 35
Total Nitrogen, TN	mg/L	120 ± 18
Total Phosphorus, TP	mg/L	12 ± 2
Total Coli Count, TCC	CFU/mg/L	6.2 × 10^6^ (6.8 log)
Most Probable Number of fecal enterococci, MPN_fe_	MPN/100 mL	1.1 × 10^4^ (4.0 log)
Total Proteolytic Count, TPC	CFU/mL	2.6 × 10^4^ (4.4 log)

* parameter value ± the measurement uncertainty for an extension factor *k* = 2; for pH ± 0.1, for COD, TOC, TN and TP the measurement uncertainty was ± 15%, for microbiological enumerations the measurement uncertainty were 0.04 log (TCC, TPC) and 0.07 log (MPN_fe_).

**Table 3:** Statistical parameters of the experiments using CCD/RSM with Statistica 13–evaluation of the effects.

Parameter	Evaluation of the Effects, COD, g O_2_/L, *R*^2^ = 0.8477, *R*^2^*_adj_* = 0.7462, 3 Parameter, 1 Block, 16 Experiments, MS = 0.0040								
	Effect	Standard Error	*p*-Value *	−95% Confidence Interval	+95% Confidence Interval	Factor	Standard Error of Factor	Lower Confidence Interval	Upper Confidence Interval
Constant Value	0.2725	0.0448	0.0002	0.1713	0.3738	0.2725	0.0448	0.1713	0.3738
pH (L)	0.0931	0.0344	0.0240	0.0153	0.1708	0.0465	0.0172	0.0077	0.1708
pH (Q)	0.0584	0.0417	0.1947	−0.0359	0.1528	0.0292	0.0209	−0.0180	0.1528
K_2_FeO_4_ (L)	0.1946	0.0344	0.0003	−0.2724	−0.1169	−0.0973	0.0172	−0.1362	−0.1169
K_2_FeO_4_ (Q)	0.1150	0.0417	0.0222	0.0207	0.2094	0.0575	0.0209	0.0103	0.2094
Time (L)	0.0383	0.0344	0.2937	−0.1160	0.0394	−0.0192	0.0172	−0.0580	0.0394
Time (Q)	0.0036	0.0417	0.9323	−0.0907	0.0980	0.0018	0.0209	−0.0454	0.0980

L-linear effect, Q-quadratic effect, * statistically significant if *p* < 0.05.

**Table 4:** Analysis of the CCD/RSM experiment using Statistica 13—verification of the adequacy of the model using ANOVA.

Parameter	Assessment of Effects, COD, g O_2_/L, *R*^2^ = 0.8477, *R*^2^*_adj_* = 0.7462, 3 Parameter, 1 Block, 16 Experiments, MS = 0.0040			
	SS	MS	F	*p* *
pH (L)	0.029567	0.029567	7.33757	0.024045
pH (Q)	0.007911	0.007911	1.96337	0.194681
K_2_FeO_4_ (L)	0.129345	0.129345	32.09914	0.000307
K_2_FeO_4_ (Q)	0.030637	0.030637	7.60318	0.022207
Time (L)	0.005011	0.005011	1.24345	0.293696
Time (Q)	0.000031	0.000031	0.00764	0.932269
Error	0.036266	0.004030	–	–

L-linear effect, Q-quadratic effect, SS-predicted residual error of sum of squares, MS-mean square error, F-statistics, * statistically significant if *p* < 0.05.

**Table 5:** Coefficients of the fitted model.

Predictor	Regression Coefficient	Standard Error	t-Value, *df* = 9	*p*-Value	−95% Confidence Interval	+95% Confidence Interval
Intercept	1.206468	0.354637	3.401978	0.007849	0.404223	2.008712
pH (L)	−0.059897	0.065887	−0.909076	0.387006	−0.208944	0.089151
pH (Q)	0.012988	0.009269	1.401202	0.194681	−0.007980	0.033957
K_2_FeO_4_ (L)	−4.423640	1.263080	−3.502263	0.006700	−7.280926	−1.566353
K_2_FeO_4_ (Q)	5.750741	2.085576	2.757387	0.022207	1.032839	10.468642
Time (L)	−0.006018	0.025262	−0.238234	0.817035	−0.063164	0.051128
Time (Q)	0.000073	0.000834	0.087398	0.932269	−0.001814	0.001960

*df*-degree of freedom.

**Table 6:** Selected physicochemical parameters of treated landfill leachate after K_2_FeO_4_, FeSO_4_ × 7H_2_O and FeCl_3_ × 6H_2_O application.

Parameter *	Unit	After K_2_FeO_4_ Application in Optimal Conditions **	After FeSO_4_ × 7H_2_O Application ***	After FeCl × 6H_2_O Application ****
		Removal, % (in Brackets) *****		
pH	–	9.0 ± 0.1	9.0 ± 0.1	9.0 ± 0.1
Chemical Oxygen Demand	mg O_2_/L	180 ± 27 (↓76.2)	475 ± 71 (↓38.1)	450 ± 68 (↓41.6)
Total Organic Carbon	mg/L	40 ± 6 (↓82.6)	145 ± 22 (↓37.0)	125 ± 19 (↓45.7)
Total Nitrogen, TN	mg/L	38 ± 6 (↓68.3)	95 ± 14 (↓20.8)	85 ± 13 (↓29.2)
Total Phosphorus, TP	mg/L	1.0 ± 0.2 (↓91.6)	0.50 ± 0.08 (↓95.8)	0.5 ± 0.08 (↓95.8)
Total Coli Count, TCC	CFU/mL	5.9 × 10^2^; 2.8 log (↓99.9)	3.5 × 10^5^; 5.5 log (↓94.4)	4.9 × 10^5^; 5.7 log (↓92.1)
Most Probable Number of fecal enterococci, MPN	MPN/100 mL	4.6 × 10^2^; 2.7 log (↓95.8)	4.6 × 10^3^; 3.7 log (↓58.2)	4.6 × 10^3^; 3.7 log (↓58.2)
Total Proteolytic Count, TPC	CFU/mL	1.9 × 10^2^; 2.3 log (↓99.3)	2.4 × 10^3^; 3.4 log (↓90.8)	2.6 × 10^3^; 3.4 log (↓90.0)

* parameter value ± the measurement uncertainty for an extension factor *k* = 2, ** in optimal conditions i.e., pH 2.3 ± 0.1, K_2_FeO_4_ 0.390 ± 0.001 g/L (= 0.111 g Fe/L) and Time 25 ± 1 min, final pH 9.0, *** FeSO_4_ × 7H_2_O 0.553 g/L (= 0.111 g Fe/L), Time 25 ± 1 min, final pH 9.0, **** FeCl_3_ × 6H_2_O 0.537 g/L (= 0.111 g Fe/L), Time 25 ± 1 min, final pH 9.0, ***** Removal = $\frac{\left(C1-C2\right)\;\times \;100\%}{C1}$ where c_1_—concentration in raw landfill leachate, c_2_—concentration in treated landfill leachate, ↓—decrease in the parameter value.
